# *UBA1* dysfunction in VEXAS and cancer

**DOI:** 10.18632/oncotarget.28646

**Published:** 2024-09-30

**Authors:** Maki Sakuma, Torsten Haferlach, Wencke Walter

**Affiliations:** ^1^MLL Munich Leukemia Laboratory, Munich, Germany; ^2^Medical Graduate Center, Technical University Munich, Munich, Germany

**Keywords:** VEXAS, MDS, clonal cytopenia, ubiquitin, inflammation

## Abstract

*UBA1*, an X-linked gene, encodes one of the only two ubiquitin E1 enzymes, playing a pivotal role in initiating one of the most essential post-translational modifications. In late 2020, partial loss-of-function mutations in *UBA1* within hematopoietic stem and progenitor cells were found to be responsible for VEXAS Syndrome, a previously unidentified hematoinflammatory disorder predominantly affecting older males. The condition is characterized by severe inflammation, cytopenias, and an association to hematologic malignancies. In this research perspective, we comprehensively review the molecular significance of *UBA1* loss of function as well as advancements in VEXAS research over the past four years for each of the VEXAS manifestations – inflammation, cytopenias, clonality, and possible oncogenicity. Special attention is given to contrasting the M41 and non-M41 mutations, aiming to elucidate their differential effects and to identify targetable mechanisms responsible for each of the symptoms. Finally, we explore the therapeutic landscape for VEXAS Syndrome, discussing the efficacy and potential of clone-targeting drugs based on the pathobiology of VEXAS. This includes azacitidine, currently approved for myelodysplastic neoplasms (MDS), novel UBA1 inhibitors being developed for a broad spectrum of cancers, Protein Kinase R-like Endoplasmic Reticulum Kinase (PERK) inhibitors, and auranofin, a long-established drug for rheumatoid arthritis. This perspective bridges basic research to clinical symptoms and therapeutics.

## INTRODUCTION

The *UBA1* (Ubiquitin-like modifier activating enzyme 1) gene, located on the X chromosome, has recently gathered significant interest within the medical community following the 2020 discovery of VEXAS (Vacuoles, E1 enzyme, X-linked, Autoinflammation, Somatic) Syndrome [1]. This novel, difficult-to-treat hemato-inflammatory disorder is caused by three somatic mutations in *UBA1*, a gene encoding for a key E1 enzyme within the ubiquitin proteasome system (UPS). These mutations, found in hematopoietic stem and progenitor cells predominantly in older males, lead to severe and refractory inflammatory symptoms and loss of mature blood cells (cytopenias). Additionally, a portion of these patients develop hematologic malignancies, including myelodysplastic neoplasms (MDS) and multiple myeloma. Notably, despite the increased risk of acute myeloid leukemia (AML) in MDS patients [2], progression to AML is extremely rare in patients with VEXAS-MDS [3].

VEXAS Syndrome captured the attention of a wide-ranging audience beyond its initial classification as a rare genetic disease, with only 28 described male patients, partly because the genetic mutation was of somatic origin with a cancer association. The perturbation of the UPS is a long-standing cause of inflammation, evidenced by multiple pediatric monogenic autoinflammatory diseases [4]. However, adult-onset genetic inflammatory diseases were not known. It is indeed surprising that loss of function of UBA1 would lead to clonal advantage, as UPS has been the target of multiple anti-cancer drugs [5–9], and UBA1 itself was identified as cancer dependency in multiple studies [10–12]. The paradoxical clonal expansion and the high incidence of MDS yet reduced AML progression in the presence of inflammation presents a unique model for exploring the intersections between inflammation, oncogenesis, and cancer resistance mechanisms.

In the four years since VEXAS was identified, screening efforts have encompassed nearly half a million individuals [13–17], revealing an estimated incidence of 1 in 4,000 among older (predominantly white) males [13]. These screenings have uncovered greater genetic and phenotypic heterogeneity within the syndrome, including variations in inflammation levels and cancer associations. This research perspective aims to delve into the phenotypic diversity of *UBA1* mutations, focusing on the impact of loss of ubiquitylation capacity on inflammogenicity, hematologic manifestations, clonality, and oncogenic potential. Based on this knowledge, research can be directed to devise therapeutic strategies tailored to the unique challenges presented by VEXAS Syndrome.

### UBA1 loss of function and VEXAS

VEXAS Syndrome results from loss-of-function mutations in *UBA1*, which encodes for a critical enzyme within the ubiquitylation pathway. UBA1, one of only two E1 enzymes, plays a foundational role in initiating ubiquitylation by activating ubiquitin [18]. This activation is a precursor event for the subsequent transfer of ubiquitin to target substrates by numerous E2 and E3 enzymes, which impart specificity to the process. Positioned at the apex of the ubiquitylation cascade, UBA1’s functionality is indispensable for the ubiquitylation of many protein substrates, implicating virtually all cellular processes in the event of its dysfunction. In fact, the consequences of *UBA1* loss-of-function mutations are profound, include embryonic lethality [19], premature death [1] and developmental defects [20] in model organisms as well as growth impairments in cell lines [21–25]. These outcomes underscore the essential role of UBA1 in cellular regulation and development.

**Figure 1:**
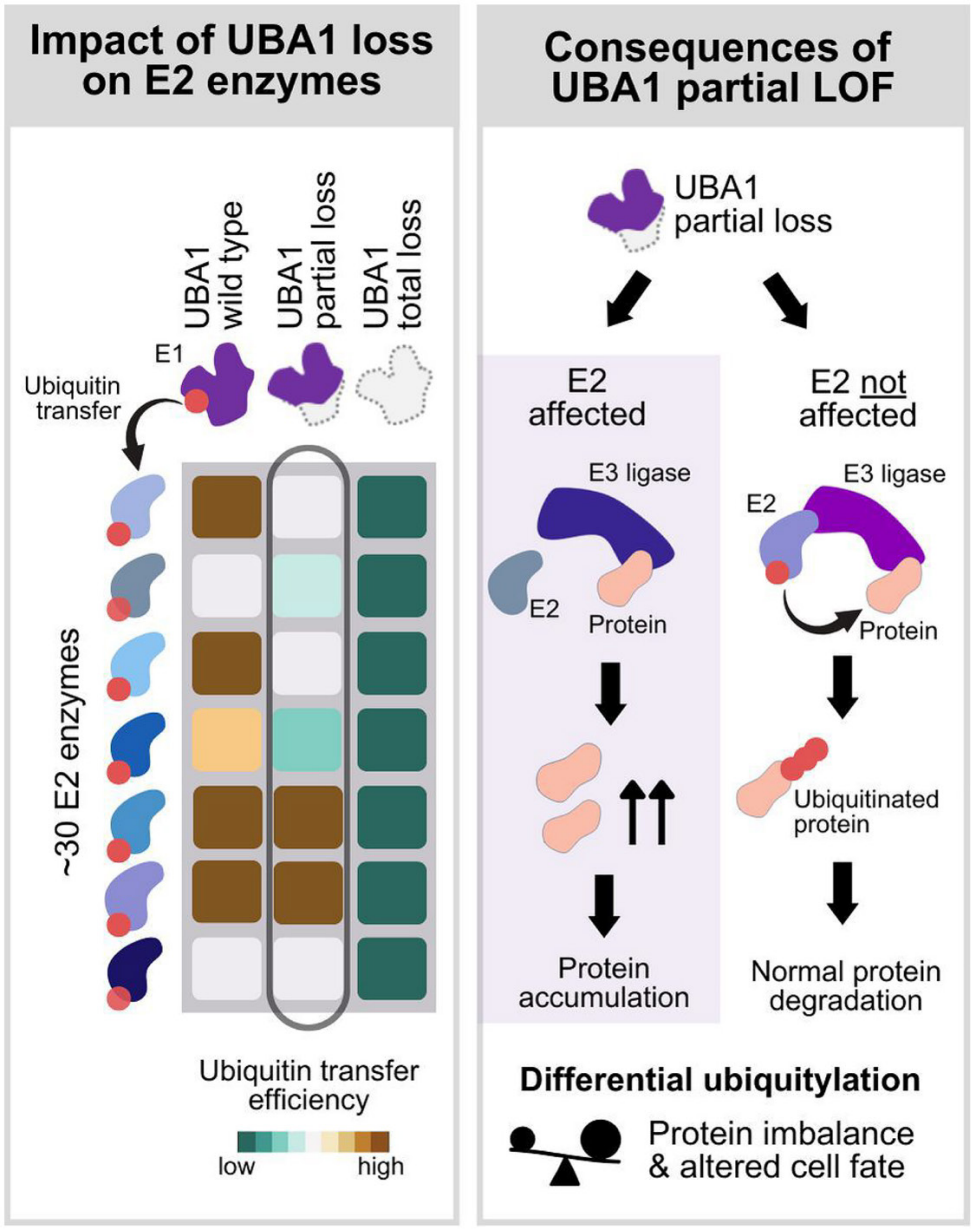
Conceptual representation of the differential effect of *UBA1* mutations based on the degree of loss of function of ubiquitin E1 enzyme UBA1. (left panel) UBA1, an E1 enzyme, activates ubiquitin and subsequently transfers the activated ubiquitin to up to approximately 30 E2 enzymes with various efficiency. The displayed heatmap illustrates the variability in ubiquitin transfer efficiency (dark green: low efficiency, brown: high efficiency) of UBA1 wild type (first column), UBA1 partial loss of function (second column) and UBA1 total loss of function (third column). Wild type UBA1 and partial loss of function mutations affect the ubiquitin transfer efficiency of a subset of E2 enzymes, whereas a total loss of function of UBA1 leads to a complete loss of loading of ubiquitin to E2 enzymes solely dependent on UBA1. (right panel) At the E2/E3-substrate transfer step, the effect of UBA1 loss of function is mediated by the decrease of available ubiquitin-loaded E2 enzymes. In the case of partial loss of function mutations, ubiquitylation of substrates can be variable due to the differential impairment of ubiquitin transfer to the E2 enzymes, which may result in imbalance of regulator proteins and altered cell fate.

*UBA1* loss-of-function mutations in VEXAS result in distinct phenotypes not observed in model organisms, including inflammation, cytopenias, thrombotic tendencies, clonality, and blood cancer associations [1]. These differences arise on the one hand from VEXAS being caused by adult-acquired somatic mutations in immune and blood cell progenitors, leading to a tissue-specific, post-developmental partial loss of function. On the other hand, VEXAS mutations are not complete loss-of-function, and the effect of partial loss of function mutations can be various (Figure 1). It has been assumed that partial loss of function mutations only affect E2 and E3 enzymes with greater reliance on UBA1 activity [24, 26, 27], which likely shifts the balance of regulatory proteins, as they ubiquitylate each other in a context-dependent way. For instance, studies in Drosophila showed that complete UBA1 loss led to apoptosis, while partial loss resulted in proliferation, due to the differential effect on the degradation of pro-apoptotic and anti-apoptotic factors [28, 29]. In HEK293T cells partial reduction of UBA1 function paradoxically increased ubiquitin-dependent import of peroxisomal proteins via a partial loss of function of a specific E2 enzyme UBE2D [30]. Research to identify the E2 and E3 enzymes most impacted by VEXAS is ongoing [31].

**Figure 2:**
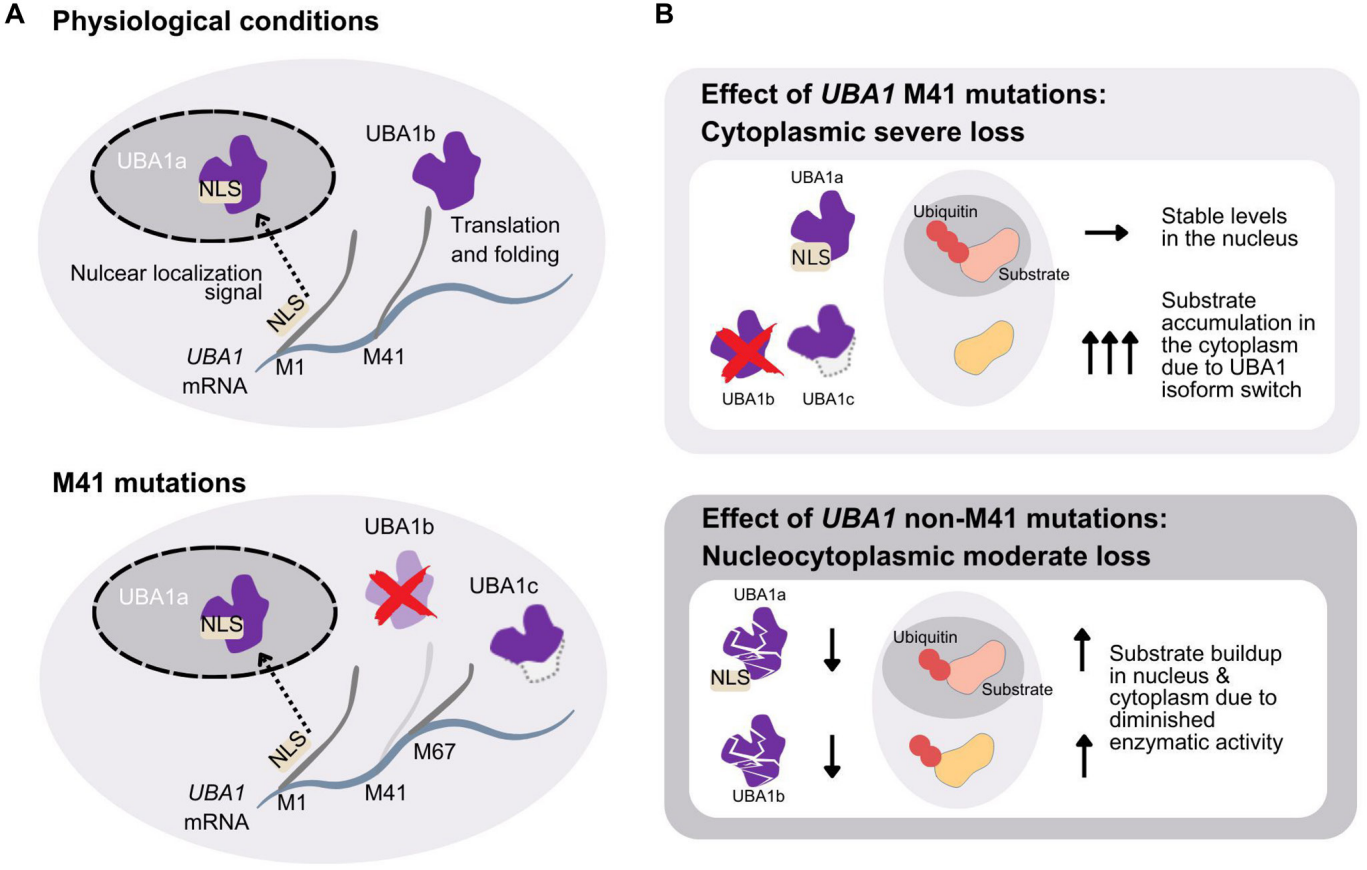
Mechanism of cytoplasmic-specific loss of function mutations and comparison with the non-M41 mutations. (**A**) *UBA1* mRNA transcript contains three alternative start codons at position M1 (UBA1a), M41 (UBA1b), and M67 (UBA1c). The transcript starting from M1 contains the nuclear localization signal (NLS) and the translated protein is transferred to the nucleus. In physiological conditions *UBA1* mRNA is also translated from position M41, lacking the NLS, and the cytoplasmic isoform UBA1b is produced (top panel). Mutations at position M41 greatly reduces the translation efficiency starting at M41 and more transcripts are translated from M67. This results in the translation product, which is the catalytically deficient cytoplasmic isoform UBA1c (bottom panel). The isoform lacks residues from M41 to A65, compared to UBA1b. (**B**) The effect of M41 mutations result in intact UBA1a in the nucleus and isoform swap in the cytoplasm of the catalytically active UBA1b to the more inactive UBA1c. This results in stable ubiquitylation in the nucleus and substrate accumulation in the cytoplasm (top panel). The effect of non-M41 mutations is equally present in UBA1a and UBA1b, respectively, and substrate accumulation should similarly be seen both in the nucleus and cytoplasm (bottom panel).

In addition, VEXAS mutations uniquely cause a cytoplasm-specific loss of UBA1 function by altering the M41 start codon of its cytoplasmic isoform [1] (Figure 2). Two protein isoforms, UBA1a and UBA1b, are produced from a single mRNA through alternative translation initiated at different start codons [32, 33]. UBA1a, starting from the M1 codon, contains a nuclear localization signal (NLS) and predominantly resides in the nucleus [34]. UBA1b, initiated from the second start codon M41, remains cytoplasmic. The ratio of UBA1a to UBA1b is physiologically regulated during cell cycle and differentiation [35, 36]. VEXAS mutations at M41 reduce UBA1b translation efficiency, favoring translation from an alternative start codon, M67, producing a catalytically inactive isoform, UBA1c [1]. Despite this, translation from M41 can still occur, with efficiency varying among mutations; M41L and M41T maintain 10-15% of wild-type protein levels, whereas M41V has only 5% [37]. It has been shown that M41V mutation significantly reduces overall poly-ubiquitylation capacity, though the nuclear isoform remains unaffected [1].

Shortly after the discovery of VEXAS, *UBA1* mutations not affecting M41 were reported in patients manifesting VEXAS-like inflammation and cytopenias [38]. One type was the splicing variants, which lead to an in-frame deletion of short exonic segments containing M41 [38–40]. The other type was, interestingly, mutations affecting functional sites in the region shared by UBA1a and UBA1b isoforms and led to a partial loss of function of both the nuclear and cytoplasmic isoforms without the appearance of the UBA1c isoform [31, 38, 41] (Figure 2). For example, a recurrent locus mutated in VEXAS patients, Y55 [14, 31, 42], has recently been shown to be the site of phosphorylation by SRC (SRC Proto-Oncogene, Non-Receptor Tyrosine Kinase), which affects ubiquitin activation efficiency [43]. The existence of non-M41 mutations suggests that VEXAS is caused by the decrease of ubiquitin activation in the cytoplasm, and the generation of UBA1c observed in M41 cases or impairment of UBA1a in the non-M41 cases are not required. However, slight phenotypic differences in inflammation, cytopenias, and associations with cancers have been observed between the M41 and non-M41 mutations [14, 44]. Furthermore, phenotypic differences among the M41 variants were also described [37, 45, 46], which suggests that the amount of residual UBA1b may affect the phenotype. The phenotypic differences of *UBA1* mutations based on UBA1b amount or defect in UBA1a may identify specific E2 or E3 enzymes responsible for each of the VEXAS symptoms.

### VEXAS manifestations and their mechanisms

**Figure 3:**
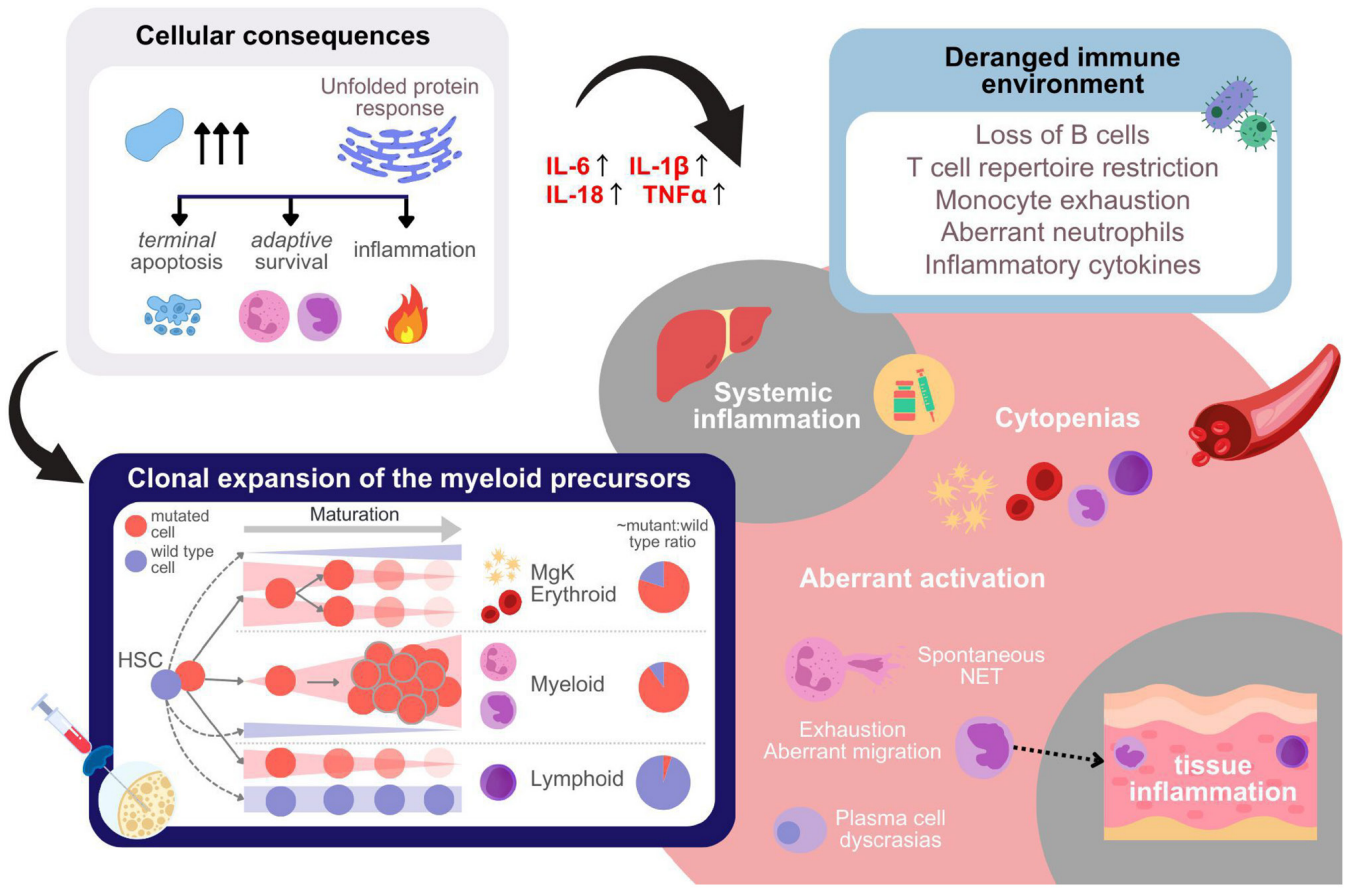
Cellular, tissue-level, immune-environmental, and systemic effect of *UBA1* mutations. *UBA1* mutations lead to substrate accumulation, which result in activation of the unfolded protein response (UPR, top left panel). This affects the cell fate of different cell types carrying the mutations in a context dependent way. In addition, UPR results in inflammatory response, including cytokine production. A list of aberrations due to mutations which may lead to altered immune microenvironment is given in the top right panel. The aberrations impair the immuno-competence of the patient. The panel at the bottom left illustrates the alterations in cell type composition within the bone marrow resulting from the cell-type-specific effects of *UBA1* mutations in hematopoietic stem cells (HSCs) and progenitor cells. Mutations in *UBA1* lead to distinct outcomes depending on the lineage of the mutated cells. Specifically, mutated cells of the lymphoid and likely also erythroid lineages progressively decrease as the cells differentiate, whereas the myeloid cells carrying the mutations undergo clonal expansion. The pie charts on the right side of the panel provide an approximate quantification of the mutant to wild type ratio per lineage observed. In peripheral blood of VEXAS patients cytopenias are observed either as a consequence of differentiation aberrations in the bone marrow, inflammatory environment, or due to cytotoxic anti-inflammatory treatment (bottom right). In addition, aberrant activation of immune cells is observed, which aggravates both systemic and tissue inflammation.

In the previous section, we provided an overview of *UBA1* mutations, the molecular implications of loss of function, and the VEXAS mutations. This section delves into VEXAS manifestations—specifically, inflammogenicity, cytopenias, clonal expansion of the myeloids, and oncogenicity—detailing their clinical and cellular characteristics and their links to reduced ubiquitylation (Figure 3), offering insights into targets of therapeutic intervention.

### Inflammogenicity

Inflammatory symptoms in VEXAS include non-infectious fever, chondritis, skin rash, and lung infiltrates [1, 46, 47]. Patients show high levels of inflammatory cytokines (IL-1β, IL-18), as well as C-reactive protein, the indicator of inflammation most widely used in the clinics [1, 48, 49]. High-dose corticosteroids are the mainstay for controlling the inflammation long-term, though their adverse effects contribute to mortality in VEXAS. Alternative anti-inflammatory treatments often fail [39], but about 30% of patients respond to JAK inhibitors like ruxolitinib and IL-6 inhibitors [50]. T-cell targeting therapies seem not as effective [39], and the inflammation seems to stem from the aberrant activation of myeloid cells, which is the predominant population carrying the mutation in the bone marrow.

Neutrophils of VEXAS patients spontaneously release neutrophil extracellular trap (NET) [1], which is inflammogenic, and monocytes of VEXAS patients aberrantly express chemokine receptors that may facilitate migration of immune cells and inflammogenicity in the skin [48]. Although the precise mechanism linking ubiquitylation impairment to the reported aberrant myeloid activation is not known, a consistent observation in VEXAS cells or cells treated with UBA1 inhibitors is the upregulation of the unfolded protein response (UPR), likely due to the decrease in the efficiency of endoplasmic reticulum-associated degradation and the consequent accumulation of misfolded proteins [4]. UPR can trigger inflammation by myriads of mechanisms, including the activation of NF-κB pathway and the inflammasome, and its dysregulation is associated with multitudes of phenotypically diverse autoimmune and autoinflammatory diseases [51, 52].

Interestingly, patients with VEXAS syndrome who have mutations other than M41 often experience less severe inflammatory symptoms [16, 44]. In fact, the effects of the UPR are not limited to inflammation but also include cell death, stress responses like reduced protein production and increased autophagy, and changes in cell differentiation [53, 54]. The phenotypic diversity observed might be linked to the different levels of proteotoxic stress caused by M41 and non-M41 mutations. Nonetheless, if the inflammation in both groups is due to the UPR in myeloid cells, focusing on the affected myeloid cells or adjusting the UPR might be more effective than targeting the wide array of cytokines and chemokines individually.

### Cytopenias

In VEXAS syndrome, there is a noticeable reduction in various blood cells [37, 47, 55], including red blood cells (98% of cases), platelets (33–54%), neutrophils (23–29%), monocytes (73%), and lymphocytes, especially B cells (91%). Anemia that requires regular blood transfusions is linked to a shorter lifespan [37], and a decrease in lymphocytes can lead to more infections among VEXAS patients, which is a leading cause of death [1]. Therefore, managing hematologic symptoms is a key component of effectively treating VEXAS patients.

Bone marrow examinations of VEXAS patients typically show a hyperplastic bone marrow with increase in myeloid progenitors and decrease in erythroid progenitors. These progenitor cells, both myeloid and erythroid, typically show characteristic vacuoles, which are likely autophagic vacuoles indicating stress [56]. Megakaryocytes (platelet progenitors) also show characteristic dysplasia [55]. About 80% of progenitor cells of all lineages carried mutations, and among mature cells, neutrophils and monocytes showed these mutations, with none found in B and T cells [1]. Single-cell studies confirmed that these mutations are present in progenitors of both lymphoid and erythro-megakaryocytic lineages [45, 57, 58]. However, there is a noticeable reduction of lymphoid cells as they develop, while the trend in erythro-megakaryocytic lineage is less evident, possibly because mature cells in this lineage don’t have a nucleus and weren’t examined, but they showed a similar pattern. In summary, the cytopenias of the lymphoid and erythro-megakaryocytic lineages seem to be due to the preferential differentiation of the hematopoietic stem cells to the myeloid lineage and/or negative selection during lymphoid and erythro-megakaryocytic differentiation (Figure 3). In contrast, neutropenia and monocytopenia are likely due to the spontaneous inflammogenic death or migration into the tissues in the periphery, as mentioned earlier.

The bone marrow differentiation bias and blood cell composition in VEXAS have been quite comprehensively described, but the *UBA1* mutation-specific molecular mechanisms that might provide insights into treatment of cytopenias are not fully investigated, partly due to the confounding hematologic side-effect of some anti-inflammatory treatment [59] and due to the general rule that controlling inflammation improves the hematologic symptoms in inflammatory diseases [60]. In fact, inflammation is known to increase granulopoiesis and decrease erythropoiesis in a multifactorial way [61, 62], and in some cases controlling inflammation improved cytopenias even without changes in *UBA1*-mutated clone size [63]. However, some patients experience worsening of their low blood cell counts during periods when inflammation is not active [64], and those with VEXAS who have mutations other than M41 may have more severe anemia but only mild signs of inflammation [44]. Interestingly, patients carrying the non-M41 mutations often have increased erythropoiesis in the bone marrow [14, 38, 42], which is unusual for anemia caused by inflammation. Thus, the intrinsic mechanism of cytopenias is plausible and worthy of investigation.

Erythropoiesis, megakaryopoiesis, and lymphopoiesis are all regulated by ubiquitylation. For instance, the receptor for erythropoietin is broken down by the E3 ligase β-TRCP [65] and RNF41 [66], both the receptor for thrombopoietin (MPL) [67] and lymphoid development factor receptor IL7R [68] by E3 enzyme CBL, and plasma cell differentiation requires UPR [69]. Most importantly, p53 is degraded by E3 enzyme MDM2, and in mice, uninhibited p53 activity by loss of Mdm2 is known to lead to bone marrow aplasia [70]. Such is relevant in VEXAS, because UBA1 inhibition has been shown to increase P53 protein level, both by chemical inhibition [9] and mutagenesis [71]. Investigation of the stability of protein regulators of hematopoiesis in VEXAS patients is an underexplored area of research, which may open new therapeutic strategies to control cytopenias in VEXAS.

### Clonal expansion of the myeloid precursors

Most treatments for VEXAS syndrome currently focus on targeting the inflammatory pathways. However, due to the short duration of success of these treatments [39], there’s an increasing interest in therapies that target the disease-causing cells themselves. By understanding what gives these abnormal cells a growth advantage in VEXAS, it might be possible to identify new druggable targets.

The variant allele fraction (VAF) of a somatic mutation can be used as a surrogate to assess clonal expansion and the VAF of UBA1 can exceed 90% in both bone marrow and peripheral blood. As mentioned, the lymphoid lineage does not contribute to the population of mutated clones nor does the erythro-megakaryocytic lineage since they are known to progressively decrease [55]. Additionally, the proportion of myeloblasts in VEXAS is usually less than 5% [47]. Efforts to create VEXAS-like cells from induced pluripotent stem (iPS) cells have been unsuccessful unless the mutation is introduced at a later stage of myeloid cell development [58]. This suggests that primarily later-stage myeloid progenitors and mature cells contribute to the clonality. However, in lab cultures, *UBA1*-mutated knock-in cell lines of the myeloid lineage do not grow well and die spontaneously [49, 58, 72], suggesting that VEXAS clonality may depend on the environment.

Recently, VEXAS patients were reported not only to be inflammatory but also immunodeficient, even after controlling for immunosuppressive treatment, either due to loss of lymphocytes or exhausted monocytes [73]. Many years before, a transposon-mediated mutagenesis experiment found transposon insertion in the intron 1 of *UBA1* to be one of the few insertion hotspots that were found in mice developing leukemia in immune-deficient but not immunocompetent mice [74]. Thus, the immunological environment created by *UBA1* mutations in the myeloid cells may favor the expansion of the mutant clones. The extrinsic aspect of clonal expansion is further supported by cases of clonal mosaicism or multiple independently arising clones. There is a case report of a patient who showed three independent *UBA1* M41-mutated clones [75], and almost every large screening attempt of symptomatic persons found at least one patient with multiple independent *UBA1*-mutated clones [16, 45], suggesting that *UBA1*-mutated clones gain advantage from the extrinsic inflammatory or immunodeficient environment. The most recent single-cell study [58] suggests that the outcome of UPR in mutated myeloid cells is the activation of an anti-apoptosis pathway, which may be one mechanism that allow the preferential survival of the mutated cells in the inflammatory milieu.

### Oncogenicity

Patients with VEXAS syndrome often receive a concurrent diagnosis of MDS and, to a lesser extent, multiple myeloma [1, 45, 55]. Understanding oncogenicity of *UBA1* mutations is crucial, especially regarding treatment strategies, because modulating ubiquitylation and inflammation can shift the balance between cell death and survival in different ways [28, 29], offering insights into potentially severe side effects. Moreover, setting aside considerations of quality of life, the median survival for VEXAS patients can reach 10 years from the first appearance of symptoms [37]. Therefore, the diagnosis of cancer would significantly affect the patient’s prognosis and overall health trajectory.

Initially MDS was reported in approximately half of VEXAS patients [46], but strict morphological evaluation of VEXAS bone marrow slides found the co-occurrence to be only 4% [55]. The confusion stems from the difficulty in distinguishing pre-malignant dysplasia from morphological changes secondary to inflammation or other non-malignant causes [76]. Other criteria for the diagnosis of neoplasms are the presence of oncogenic mutations. However, two large studies [16, 45] showed that VEXAS patients rarely harbor co-mutations other than *DNMT3A* or *TET2*, which are often also detected in healthy elderly individuals [77]. Thus, the exact prevalence of MDS among VEXAS patients, in the strictest definition, remains uncertain. Additionally, the presence of an MDS diagnosis alongside VEXAS does not appear to influence patient survival rates [37]. This observation challenges the conventional understanding of malignancy, as one would expect a cancer diagnosis to affect survival outcomes. Consequently, the link between MDS and VEXAS does not advocate for the oncogenicity of *UBA1* mutations.

The co-diagnosis of multiple myeloma is more difficult to interpret. Due to the absence of *UBA1* mutations in lymphocytes and the demographic overlap, some believe that multiple myeloma develops independently of *UBA1* mutations [55]. However, the incidence of multiple myeloma in European males over 50 years is approximately 0.03% [78]. The prevalence would be no more than 0.3%, whereas in VEXAS the co-diagnosis is 3–8%. Further research in plasma cells is necessary to understand this association.

Currently, the association between *UBA1* mutations and cancer remains uncertain, yet there is a growing body of literature on the connection between non-M41 *UBA1* mutations and various cancers. UBA1 is a known orchestrator of DNA damage response [79, 80], and coinciding with the discovery of VEXAS syndrome, *UBA1* mutations were implicated as potential key factors in the development of lung cancer among non-smokers, identified through advanced bioinformatics methods [81]. The patients were all females. None of them harbored the M41 mutations, and instead frameshift, nonsense, and non-M41 missense mutations. In addition, we reported that somatic non-M41 variants are detected in various hematologic neoplasms, including lymphoid malignancies [14]. The pathogenicity of the variants is not confirmed, but the possibility that different degrees of loss of function mutations of *UBA1* may have oncogenic potential is worth exploring to design safe therapy.

### Modifiers of the phenotype – age, sex, and cell type

In the previous section, we mentioned that frameshift and non-sense mutations in lung cancer were exclusively observed in female patients. *UBA1* is a known X chromosome escape gene, and studies consistently find *UBA1* to be expressed approximately 1.2-fold higher in the peripheral blood of females than males [82–85]. *UBA1* pathogenic mutations show a clear sex bias in VEXAS [1, 14, 46] and X-linked spinal muscular atrophy (XL-SMA), a congenital neuromuscular disease caused by germline *UBA1* mutations [86]. The functional impairment of UBA1 caused by mutations associated with XL-SMA is relatively minor when compared to VEXAS syndrome [31]. Female carriers of XL-SMA pathogenic *UBA1* mutations are asymptomatic, but a female child with *UBA1* gene deletion is affected [87]. Thus, the baseline UBA1 expression and the extent to which mutations impair its function appear to influence the resulting phenotype. Moreover, UBA1 protein expression decreases with age in mouse brains [88], and researchers of neurodegenerative diseases propose a threshold hypothesis where disease onset occurs once ubiquitylation capacity falls below a critical functional threshold [89]. Age might also play a role in VEXAS syndrome, as evidenced by the identification of several younger, asymptomatic patients carrying the *UBA1* M41 mutations, who exhibited lower VAFs [15]. It appears that both age and sex influence the overall capacity for ubiquitylation, which may potentially impact the manifestation and progression of the disease [14]. Furthermore, the neuron-specific phenotype linked to germline mutations suggests varying thresholds of UBA1 functional deficiency across cell types. Protein aggregates, a known cause of neurodegenerative diseases, indicate neurons’ particular vulnerability to UBA1 dysfunction, potentially exacerbated by impaired direct interactions with proteins crucial for neural development (e.g., SMN1 [90, 91], Gigaxonin [92]).

### Novel treatment strategies for VEXAS

**Figure 4:**
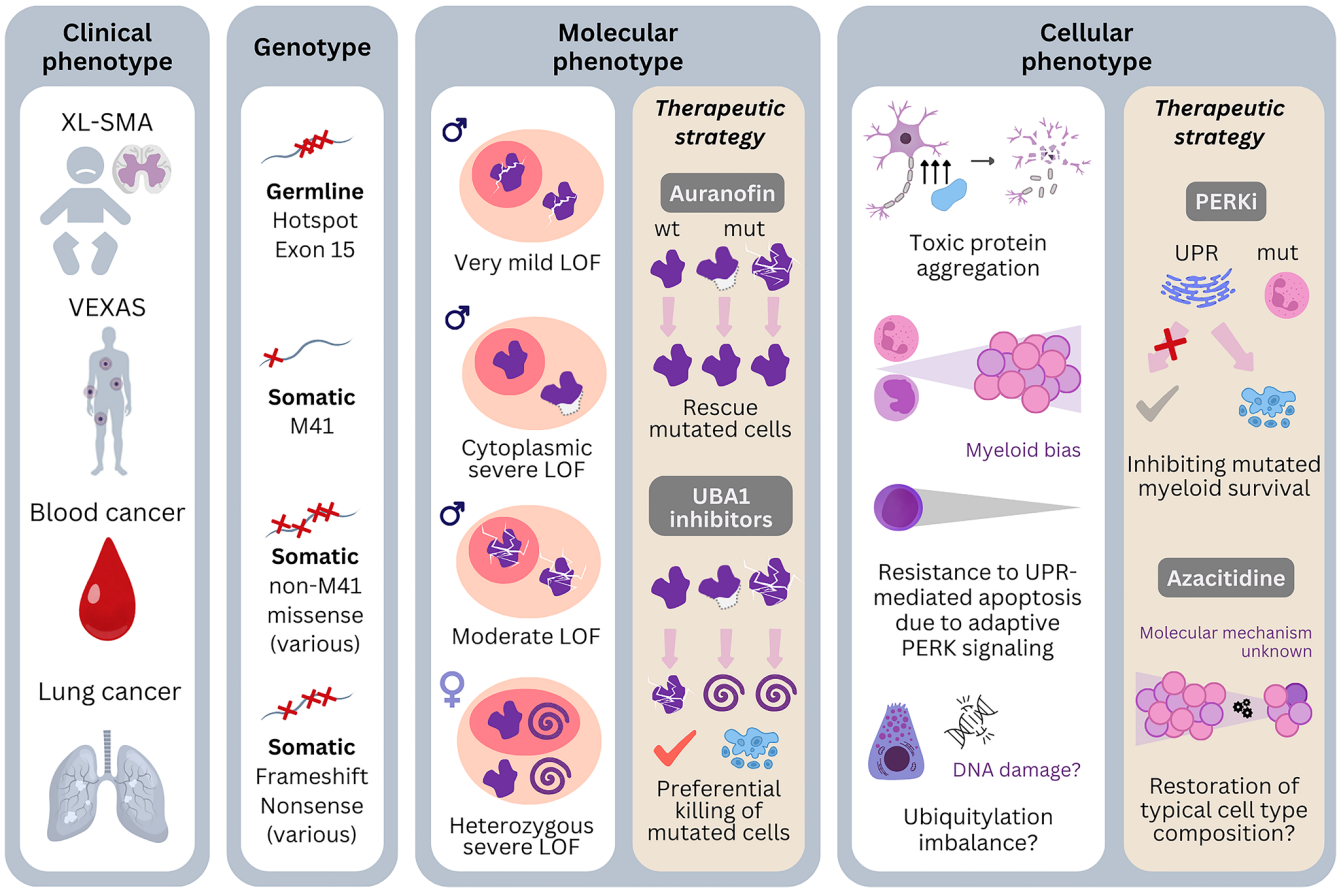
Genotype-phenotype associations of pathogenic *UBA1* mutations and possibilities of therapeutic targeting. Four different clinical phenotypes of pathogenic *UBA1* mutations are known: X-linked spinal muscular atrophy (XL-SMA) is caused by germline mutations which have hotspot in exon 15. VEXAS is caused by somatic mutations in the cells of the bone marrow. M41 is the most frequent genotype but recurrent non-M41 mutations are also reported. Blood cancers are associated with both M41 and non-M41 mutations. Lung cancer is also reported with *UBA1* mutations in females, which include frameshift and nonsense mutations. Molecularly, the degree of the enzymatic dysfunction and alteration of localization are different by genotypes. Therapies targeting this level: Auranofin tries to ameliorate phenotype by improving enzymatic dysfunction, whereas UBA1 inhibitors targets to tip the balance of survival to apoptosis by preferentially in cells with severe dysfunction. The cellular phenotypes of the mutations not only depend on the nature of the mutations but also on the affected cell type. Neuronal cells are particularly sensitive to protein aggregates and the mutations may be toxic with only slight enzymatic dysfunction. Other phenotypic mechanisms may be binding defects to proteins important in neural development. Concerning VEXAS, myeloid cells seem to be more resistant to UPR-mediated apoptosis due to activation of the PERK arm of the UPR. Therapies targeting of this level: PERK inhibitors try to prevent the preferential escape from apoptosis of the myeloid cells. Azacitidine likely also restore the cell type composition, but the exact mechanism is not known. The diverse clinical phenotypes are likely associated with the variety of mutations and their nature of loss of function and affected cell types. Abbreviations: wt: wild type; mut: mutated; LOF: loss of function; UPR: unfolded protein response; i: inhibitors.

The standard approach to treating VEXAS syndrome starts with administering high-dose corticosteroids, followed by a range of anti-inflammatory medications to gradually reduce the corticosteroid dosage. Additionally, supportive care is provided to manage cytopenias, infection, and thrombotic tendency. However, our review of research on the molecular and cellular impacts of *UBA1* mutations highlights critical vulnerabilities in VEXAS pathogenesis (Figure 4). Therapies aimed directly at targeting the disease-causing clones could potentially offer more effective relief from all the VEXAS symptoms. Below, we detail several clone-targeting drugs and their mechanisms of action.

### Azacitidine – immunomodulatory effects?

Azacitidine emerged as a first candidate for effective treatment, supported by prior evidence of some success in managing hematoinflammatory symptoms in patients with a co-diagnosis of MDS and systemic inflammatory diseases [93, 94]. Given its approval for MDS, more VEXAS patients with a co-diagnosis of MDS receive Azacitidine, with some studies indicating that patients who respond to the treatment often have concurrent mutations in *DNMT3A* [95]. Beyond its primary effects, hypomethylating agents are noted for their immunomodulatory properties, such as diversifying T cell repertoire [96, 97]. VEXAS T cells are clonally restricted [57], and this ability to modify the cellular environment, potentially hindering the clonal expansion of *UBA1* mutations, represents another avenue through which it may exert its therapeutic effects. Early results from a Phase II clinical trial involving VEXAS patients have been promising [98] and a case has been reported in which Azacitidine effect extends after cessation of therapy [99].

### UBA1 inhibitors – synthetic lethality

UBA1 inhibitors were originally developed for cancer treatment, based on the premise that cancer cells require more ubiquitylation compared to normal cells [12]. In healthy physiological conditions, activated ubiquitin exists in abundance, suggesting that UBA1 inhibition might not significantly impact normal cellular functions [18]. However, in the context of VEXAS syndrome, the situation is different. There is an observed decrease of about 90% in UBA1b protein levels, potentially making these cells more vulnerable to UBA1 inhibition. Building on this theory, Chiaramida et al. [49] administered a UBA1 inhibitor TAK-243 to a cell line model knocked-in with M41L mutation and showed that VEXAS cells are killed at a lower concentration than the parent cell line, indicating a potential therapeutic window.

The question of whether there are differences in response to UBA1 inhibitors between M41 variants and non-M41 variants remains unanswered. Variability in response to UBA1 inhibitors has been noted among cell lines even within the same cancer. In squamous cell carcinoma, cell lines with lower expression of UBA1 responded better to TAK-243 [100], whereas in glioblastoma cell lines with lower expression of the ER chaperone GRP78 and not UBA1 expression responded better to TAK-243 [101]. The factors predicting treatment response in VEXAS syndrome have yet to be identified, though sex, age, and specific genetic mutations are potential sources of variability. Particularly, the non-M41 variants may respond differently due to their impact on the nuclear isoform of UBA1. The initial study in TAK-243 [9] indicates that its cytotoxic effects are mediated through several mechanisms, including DNA damage response and the impaired degradation of key proteins such as p53. These proteins play a crucial role in triggering cell cycle arrest and apoptosis in the presence of irreversible DNA damage [9], which seems more relevant in the nucleus.

The nuclear isoform is particularly prominent in G1 and G2 phases in HeLa cells [36], so the cell types which are often cycling are likely to be more sensitive. Further research is necessary to understand the effect of UBA1 inhibitors in the bone marrow, comparing the different M41 variants as well as the non-M41 variants. Combination therapies have been attempted in other cancers, such as radiotherapy and PARP1 inhibitors [102], which can be another direction of investigation. An additional note is that one of the UBA1 inhibitors, PYR-41 activates sumoylation at the same time, because ubiquitylation and sumoylation target the same residues of overlapping target substrate [103].

### PERK inhibitors – UPR modulation

UPR modulation is one of the mechanisms that may alter survival advantages of *UBA1*-mutated myeloid cells. Ganesan et al. showed that *UBA1*-mutated myeloid cells gain survival advantage over wild type cells by activating the PERK-ATF4 arm of UPR [58]. In a M41V knock-in iPSC model, they showed that the M41V cells were more sensitive to PERK inhibitor GSK2606414 than the wild type cells. More preclinical studies are awaited to develop this promising target.

### Auranofin – improving defective UBA1 function

A novel strategy in VEXAS therapy came from an observation that UBA1c can be reactivated by Auranofin, a long-established drug for rheumatoid arthritis. Auranofin was found to enhance UBA1 binding to 20 out of 36 E2 enzymes tested and improved polyubiquitylation of multiple substrates [104]. Importantly, the effective dose was 4.5 to 73 times lower than the approved maximum therapeutic concentration for rheumatoid arthritis. Auranofin shows cytotoxic effect to chronic lymphocytic leukemia [105] as well as chronic myeloid leukemia [106], and its effect in VEXAS cells needs to be investigated.

## CONCLUSIONS

VEXAS is a disease caused by *UBA1* mutations in hematopoietic stem and progenitor cells. VEXAS phenotypes include inflammation, cytopenias, thrombotic tendency, clonality and potential oncogenicity. These diverse clinical features arise from the effects of *UBA1* mutations across distinct cell types within the bone marrow and peripheral blood. The relationship between specific *UBA1* genotypes and the resultant phenotype appears to be modulated by factors including age, sex, and the cellular context. Future studies clarifying how the genotype and host factors, which determine the severity and localization of the loss of function of UBA1, change the immune environment and shape the clinical phenotypes will be crucial. This, in turn, is expected to inform the development of targeted therapeutic interventions. The advent of clone-targeting therapies offers a promising avenue, yet a more detailed understanding of the specific E2/E3 enzymes involved and the differential impact of the UPR across cell types may identify novel therapeutic targets. Ultimately, a thorough grasp of the pathogenesis of VEXAS, from genetic mutations to clinical manifestations, will be pivotal in devising safe and effective therapeutic strategies to fight this challenging disease.
